# MicroRNA-212-3p inhibits the Proliferation and Invasion of Human Hepatocellular Carcinoma Cells by Suppressing CTGF expression

**DOI:** 10.1038/s41598-019-46088-w

**Published:** 2019-07-08

**Authors:** Jian-qing Chen, Yang-liu Ou, Zhi-ping Huang, Yong-gang Hong, Yuan-ping Tao, Zhen-guang Wang, Jun-sheng Ni, Li-qiang Hao, Hui Lin

**Affiliations:** 1 Department of Digestive Internal, Shidong Hospital, Yangpu District, Shanghai, Anhui Medical University, 999 Shiguang Road, Shanghai, 200438 China; 2 The Third Department of Hepatic Surgery, Eastern Hepatobiliary Surgery Hospital, Second Military Medical University, 225 Changhai Road, Shanghai, 200438 China; 3 Department of Hepatobiliary Surgery, General Hospital of Southern Theatre Command, Guangzhou, 510010 China; 4 Department of Colorectal Surgery, Changhai Hospital, 168 Changhai Road, Shanghai, 200433 China; 5 The First Department of General surgery, Shidong Hospital, Yangpu District, Shanghai, Anhui Medical University, 999 Shiguang Road, Shanghai, 200438 China

**Keywords:** Cancer, Diseases

## Abstract

MicroRNA-212-3p inhibits several human cancers but its effects on hepatocellular carcinoma (HCC) remain unclear. In this study, we show that miR-212-3p is down-regulated in HCC cell lines and tissues, and correlates with vascular invasion (p = 0.001), and the absence of capsule formation (p = 0.009). We found that miR-212-3p influenced the epithelial to mesenchymal transition (EMT) of HCCLM3 and Huh7 cells. Mechanistically, miR-212-3p repressed cell invasion through the suppression of connective tissue growth factor (CTGF). We therefore validate the anti-HCC effects of miR-212-3p through its ability to suppress CTGF and subsequent EMT.

## Introduction

Hepatocellular carcinoma (HCC) is the second primary cause of cancer mortality, and the sixth most common malignancy worldwide^1,2^. Although the survival of HCC patients had improved due to improved diagnosis, efficient monitoring, and several treatment alternatives, its long-term survival remains poor. HCC-related deaths occur from a high metastasis and recurrence probability after surgical resection^3^, the underlying molecular mechanisms of which have not been established.

Epithelial-mesenchymal transition (EMT) is a key metastatic process in which cells lose their polarity and cell-cell adhesion, and gain migratory and invasive phenotypes to become mesenchymal stem cells^4–6^. Epithelial cells migrate and invade under the influence of EMT, migrating from the original tumor site and invading the vascular or lymphatic systems^7^. A complex HCC cells network controls EMT. Recent studies have demonstrated that microRNAs are critical regulators of these processes^8,9^.

MicroRNAs (miRNAs) are single-stranded non-coding RNAs that affect several biological activities, including cell development, differentiation, and carcinogenesis^10^. They bind to the 3′-untranslated region (3′-UTR) of cellular RNAs and regulate tumor suppressor or oncogene expression^11^. The 17p13.3 site miR-212-3p can inhibit glioblastoma proliferation via SGK3, and control HCC cell migration and invasion via YAP1^12^. Additionally, miR-212 suppresses XIAP to inhibit renal cell cancer proliferation and invasion, whilst functioning as a tumor suppressor through SOX4, in non-small cell lung cancer (NSCLC)^13,14^. The clinical significance of miR-212-3p in HCC has yet to be defined.

In this study, we demonstrate the downregulation of miR-212-3p expression in HCC cell lines and tissues. The loss of miR-212-3p correlated with tumor encapsulation deficiency and vascular invasion in HCC. We found that restoring miR-212-3p expression could prevent HCC cell invasion. At the molecular level, CTGF was identified as a direct target of miR-212-3p revealing new insights into the suppression of HCC via a CTGF-mediated EMT process.

## Materials and Methods

### Clinical samples and ethics statement

This study was approved by the ethic committee of Eastern Hepatobiliary Surgery Hospital. This included 83 HCC patients, diagnosed pathologically between January 2010 and December 2013, in the Eastern Hepatobiliary Surgery Hospital (Shanghai, China). The patients received no preoperative therapy. Samples were collected, immediately frozen in liquid nitrogen, and stored at −80 °C. All HCC and adjacent non-cancerous tissues (ANT) were simultaneously harvested. The hospital’s Institutional Review Board approved the study in strict adherence to the Declaration of Helsinki protocol. Written informed consent was requested from each patient. Post-surgical follow-up of all patients involved procuring survival information through telephone contact.

### Cell culture and transfection

Immortalized human hepatocyte LO2 cells, and human HCC cells (HCCLM3, HUH7, Hep3B, andHepG2) were purchased from the Shanghai Institute for Cell Biological Science (Shanghai, China). Cells were cultured in Dulbecco’s modified Eagle’s medium (DMEM) supplemented with 10% fetal bovine serum (FBS), 100 U/ml penicillin, and 100 mg/ml streptomycin (Gibco; Thermo Fisher Scientific, Inc., Waltham, MA, USA).

The Shanghai GenePharma Co., Ltd. (Shanghai, China) synthesized the negative control (NC) miRs and miR-212-3p mimics. For CTGF overexpression, CTGF coding oligonucleotides were inserted into pcDNA3.1 (Invitrogen, Shanghai, China). Huh7 and HCCLM3 cells (6 × 10^4^ cells per well) were seeded into 24-well plates and transfected with 50 nM miR-212-3p mimics or NC, and pcDNA-CTGF or empty vector using Lipofectamine^®^2000 (Invitrogen, Shanghai, China). Cells were harvested for analysis 48 h post-transfection.

### RNA extraction, reverse transcription, and quantitative real-time PCR (qRT-PCR)

RNA extraction, reverse transcription and quantitative real-time PCR (qPCR) were performed as previously described (20). Primer sequences are listed in Table 1.

**Table 1:** Primer sequences used in the study.

U6	Forward: 5′-CTCGCTTCGGCAGCACA-3′
	Reverse: 5′-AACGCTTCACGAATTTGCGT-3′
CTGF	Forward: 5′-ACGGATTTGGTCGTATTG-3′
	Reverse: 5′-GGAAGATGGTGATGGGATT-3′
E‐cadherin	Forward: 5′-TACACTGCCCAGGAGCCAGA-3′
	Reverse: 5′-TGGCACCAGTGTCCGGATTA-3′
ZO-1	Forward: 5′-CCAGCAAAGCAATCCCCACT-3′
	Reverse: 5′-ATGGTGCCGGAGTTGATTGC-3′
Fibronectin	Forward:5′-TCGAAAGATCCGCAGCACAT-3′
	Reverse: 5′-TGGTGTAGGTCGGAGGAAGC-3′
N-cadherin	Forward: 5′-TCAGGCGTCTGTAGAGGCTT-3′
	Reverse: 5′-ATGCACATCCTTCGATAAGACTG-3′
GAPDH	Forward: 5′-TGTGGGCATCAATGGATTTGG-3′
	Reverse: 5′-ACACCATGTATTCCGGGTCAAT-3′

### Transwell migration and invasion assays

Transwell migration and invasion assays were performed as previously described (20).

### Western blot analysis

Cells were lysed in RIPA buffer and proteins were separated on SDS-PAGE gels. Proteins were transferred onto polyvinylidene difluoride (PVDF) membranes which were blocked for 1 h in 5% non-fat milk in TBST (10 mM Tris, pH 8.0, 150 mM NaCl, 0.5% Tween 20) at room temperature. Membranes were washed in TBST and probed overnight at 4 °C with one of the following primary antibodies (obtained from Cell Signaling Technologies, Danvers, MA, USA unless otherwise stated): E-cadherin (ab1416, 1:500; Abcam, Cambridge, MA, USA), CTGF (ab6992, 1:500; Abcam, Cambridge, MA, USA), ZO-1 (ab96587, 1:500; Abcam, Cambridge, MA, USA), N‐cadherin (ab213756, 1:500; Abcam, Cambridge, MA, USA), Fibronectin (ab2413, 1:500; Abcam, Cambridge, MA, USA), and GAPDH (1:1000, ab181602; Abcam, Cambridge, MA, USA). Membranes were washed in TBST and incubated for 2 h with HRP-conjugated secondary antibodies at room temperature. Proteins were visualized using the ECL system (Beyotime, Jiangsu, China).

### Cell counting kit-8 (CCK-8) proliferation assays

CCK-8 kits (Solarbio Biotechnology, Beijing, China) were used to measure Huh7 or HCCLM3 cells proliferation. Cells were seeded at a density of 5 × 10^3^ cells/well into 96-well plates and incubated for 48 h. CCK-8 reagent (10 µl) was added to each well for 1 hr. Miniature microplate readers were used to determine the OD values at 450 nm. Experiments were performed in triplicate.

### Luciferase reporter assays

TargetScan (http://www.tar getscan.or g/v ert_72/) was used to identify CTGF as a target gene for luciferase reporter assays and miR‐212-3p correlation analysis. Mutated (MUT) or wild-type (WT) CTGF 3′‐UTRs were ligated into pGL3 (Promega, Madison, WI, USA). The luciferase reporter comprised CTGF 3′-UTR regions with miR-212-3p targets or mutant site indicators, to determine whether miR-212-3p directly targeted CTGF. Cells were seeded in triplicate into six-well plates, and left to settle for 12 h. Cells were transfected with miR-212-3p or NC controls (Thermo Fisher Scientific) and Dual-Luciferase Reporter Assays (Promega Corporation, Fitchburg, WI, USA) were employed 24 h post-transfection.

### Statistical analyses

SPSS 17.0 (SPSS Inc., Chicago, IL, USA) was used for statistical analyses. Data are shown as the mean ± SD. A student’s t-test was used to statistically differentiate the groups. A one-way ANOVA was employed for multiple tests. The correlation of clinic pathological characteristics with miR-212-3p expression was analyzed via the chi-square test. The Kaplan Meier method was used to plot survival curves, which were compared using the log-rank test. Spearman correlation analysis evaluated the correlation between miR-212-3p and CTGF in HCC tissues. A P < 0.05 was deemed significant. All experiments were performed in triplicate.

## Results

### HCC cell lines and tissues display reduced miR-212-3p expression

**Figure 1:**
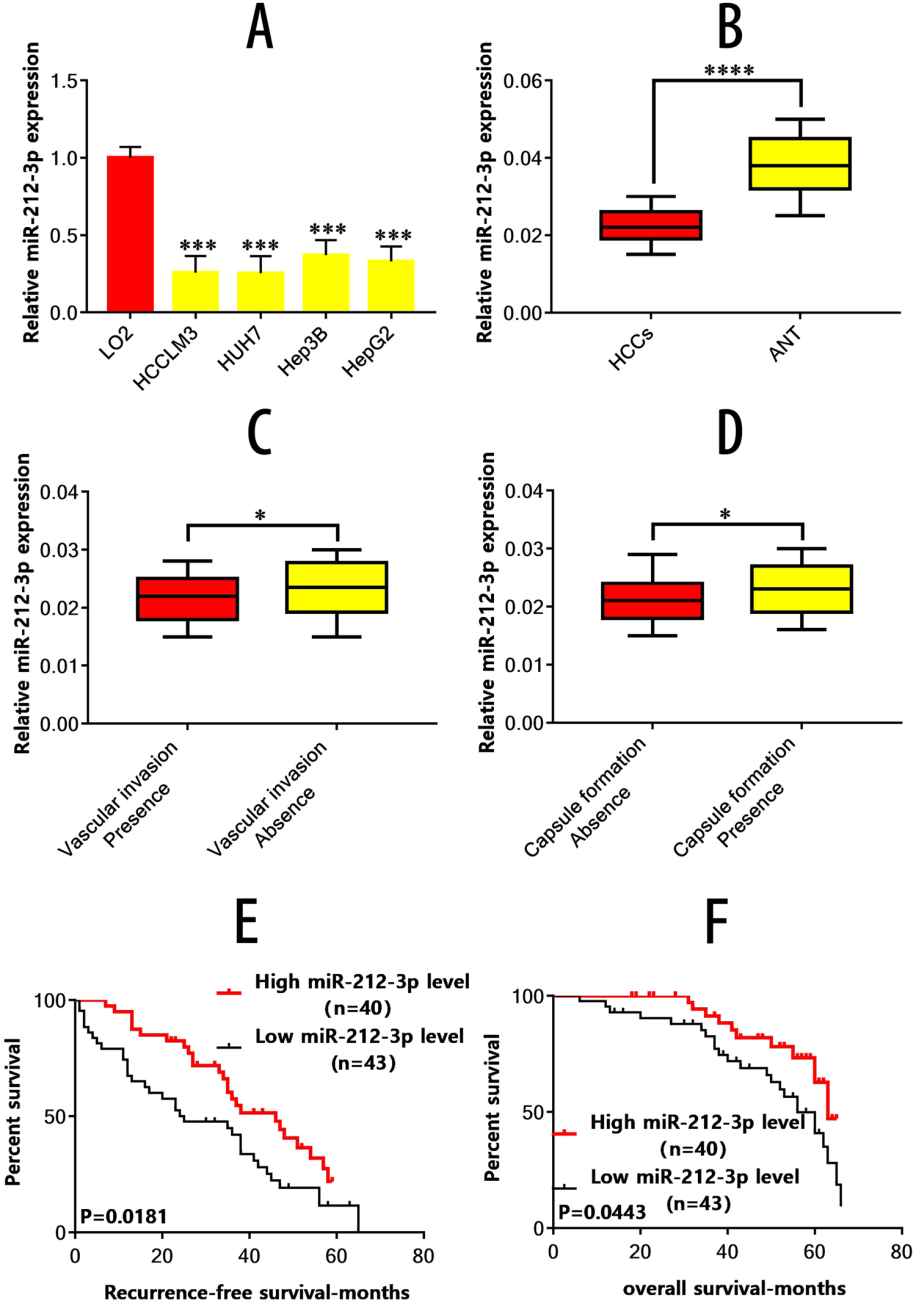
miR-212-3p is down-regulated in HCC and is associated with poor prognosis. (**A**) Expression of miR-212-3p in the HCC cell lines HCCLM3, Huh7, Hep3B, and HepG2 is significantly lower than the immortal liver cell line L02. (**B**) Expression levels of miR-212-3p in 83 paired HCC tissues and ANT were analyzed by qRT-PCR. (**C**) miR-212-3p is downregulated in HCC tissues with vascular invasion compared with those without vascular invasion. (**D**) miR-212-3p is downregulated in HCC tissue lacking tumor encapsulation compared to those with tumor encapsulation. (**E,F**) Low miR-212-3p expression is associated with a shorter RFS (**E**) and OS (**F**) of HCC. *P < 0.05; **P < 0.01; ***P < 0.001.

The expression of miR-212-3p in the HCC cell lines Huh7, HCCLM3, HepG2 and Hep3B was assessed by quantitative real-time polymerase chain reaction (qRT-PCR) and compared to the immortalized human hepatic cell line LO2. The expression of miR-212-3p was considerably lower in HCC cell lines (Fig. 1A). Subsequently, HCC tissues and ANT were assessed for miR-212-3p expression. The levels of miR-212-3p were considerably lower in 83 paired HCC tissues, in comparison to the corresponding ANT tissue (Fig. 1B). In addition, qRT-PCR assays were used to substantiate vascular invasion and tumor encapsulation deficiency following miR-212-3p down-regulation (Fig. 1C,D). This indicated that miR-212-3p could suppress HCC tumorigenesis and invasion.

### Low miR-212-3p expression indicates poor prognosis in HCC

The correlation between miR-212-3p expression and the clinic pathologic aspects of HCC were further studied. The relative median values of the 83 HCC samples were classed as low or high miR-212-3p expression using defined cut-off values. As per results, low miR-212-3p expression was linked to a lack of capsule formation (p = 0.009), and the presence of vascular invasion (p = 0.001), (Table 2). Recurrence-free survival (RFS) and overall survival (OS) were comparatively higher in the high miR-212-3p expression group (Fig. 1E,F) assessed via Kaplan-Meier survival analysis. This suggested that miR-212-3p expression is related to poor HCC prognosis, and is a potential prognostic marker for HCC stratification.

**Table 2:** Correlation between miR-212-3p expression and the pathological variables of the 83 HCC cases.

Clinic pathologic variables	n	miR-212-3p		P-value
		High (n = 40)	Low (n = 43)	
**Gender**				0.214
Male	59	31	28	
Female	24	9	15	
**Age (Years)**				0.131
≤60	67	35	32	
>60	16	5	11	
**AFP (ng/ML)**				0.158
≤20	39	22	17	
>20	44	18	26	
**Hepatitis B status**				0.199
Negative	7	5	2	
Positive	76	35	41	
**Liver Cirrhosis**				0.580
Absence	23	13	10	
Presence	60	27	33	
**Liver function**				0.428
Child-Pugh A	73	36	37	
Child-Pugh B	10	4	6	
**Tumor size (cm)**				0.165
≤5	31	18	13	
>5	52	22	30	
**Tumor nodule number**				0.074
Solitary	52	29	23	
Multiple(≥2)	31	11	20	
**Capsule formation**				0.009
Presence	48	29	19	
Absence	35	11	24	
**Edmondson-Steiner grade**				0.739
I-II	42	21	21	
III-IV	41	19	22	
**Vascular invasion**				0.001
Absence	43	28	15	
Presence	40	12	28	
**TNM stage**				0.863
Stage I	34	16	18	
Stage II-III	49	24	25	
**BCLC stage**				0.151
Stage 0-A	39	23	16	
Stage B-C	44	19	25	

AFP alpha-fetoprotein, TNM tumor node metastasis, BCLC Barcelona Clinic Liver Cancer.

### Up-regulation of miR-212-3p inhibits HCC cell invasion

**Figure 2:**
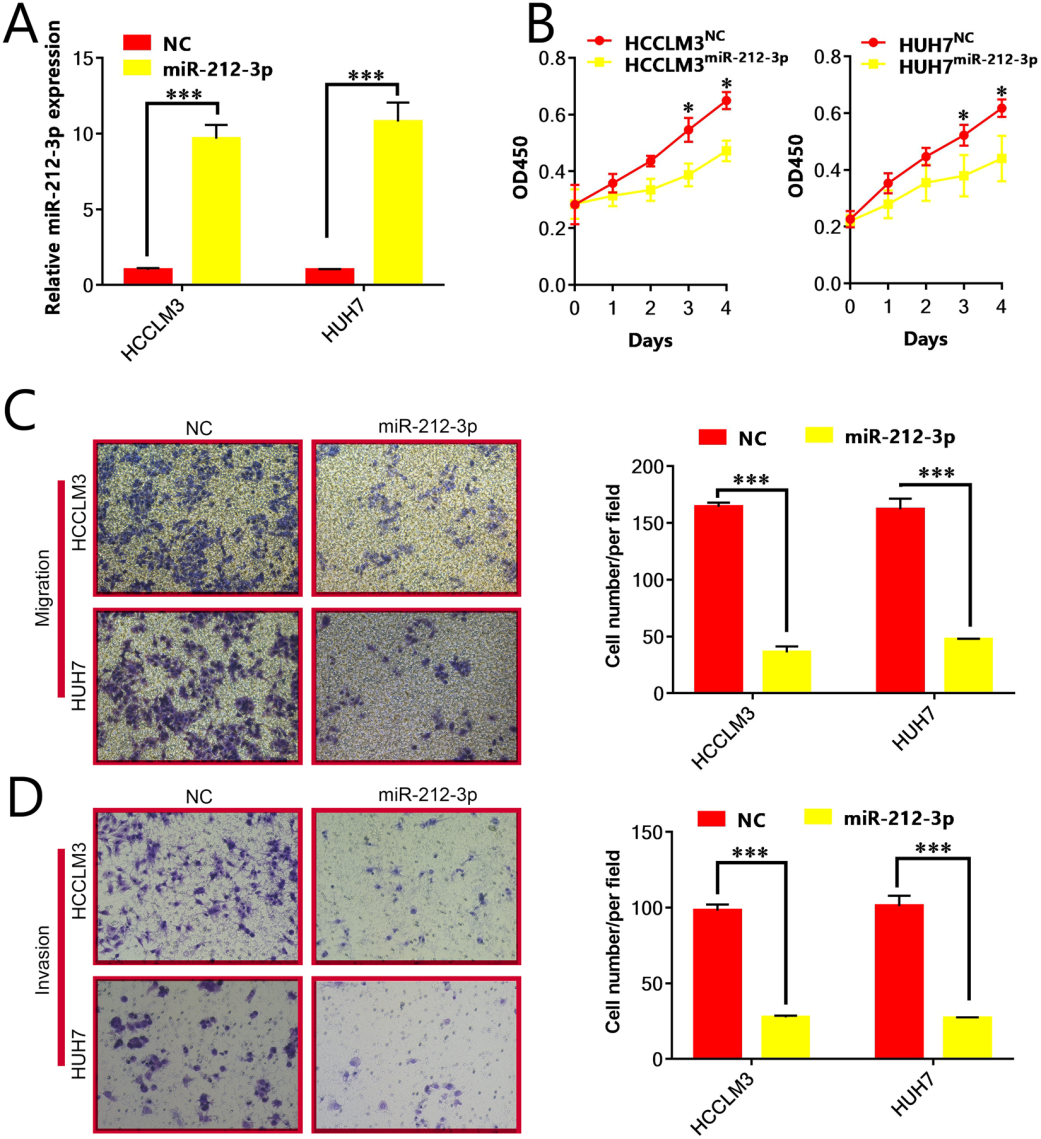
Upregulation of miR-212-3p inhibits the proliferation, migration, and invasion of hepatocellular carcinoma cells. Transfection with miR-212-3p significantly increases the expression of miR-431-5p in (**A**) HCCLM3 and Huh7 cells. (**B**) CCK-8 assays were used to examine the proliferation of HCCLM3 and Huh7 cells at 0, 24, 48, 72, and 96 hours post-transfection with miR-212-3p. (**C**) Transwell assays revealed that the upregulation of miR-212-3p significantly downregulated the number of HCCLM3 and Huh7 cells that migrated through the membrane compared to the NC group. (**D**) Transwell assays revealed that the upregulation of miR-212-3p significantly downregulates the number of HCCLM3 and Huh7 cells that invaded through the membrane compared to the NC group. 200× magnification, n = 3, *P < 0.05; **P < 0.01; ***P < 0.001.

Functional analysis was used for the design of ectopic miR-212-3p constructs that were transfected into HCC lines (HUH7^miR-212-3p^ and HCCLM3^miR-212-3p^). qRT-PCR confirmed successful miR-212-3p expression (Fig. 2A). We first examined the effects of miR-212-3p on Huh7 and HCCLM3 proliferation via CCK-8 assays. Compared to the control group (Fig. 2B), the overexpression of miR-212-3p significantly reduced cell proliferation, suggesting that miR-212-3p expression correlates with HCC proliferation.

We next assessed the migration and invasion of miR-212-3p expressing HCC cells via transwell assays. As shown in Fig. 2C,D, enhanced miR-212-3p expression reduced the invasiveness of Huh7 and HCCLM3 cells compared to NC groups. These data confirm that miR-212-3p inhibits HCC proliferation, migration, and invasion.

### Up-regulation of miR-212-3p alters the expression of EMT markers in HCC cells

**Figure 3:**
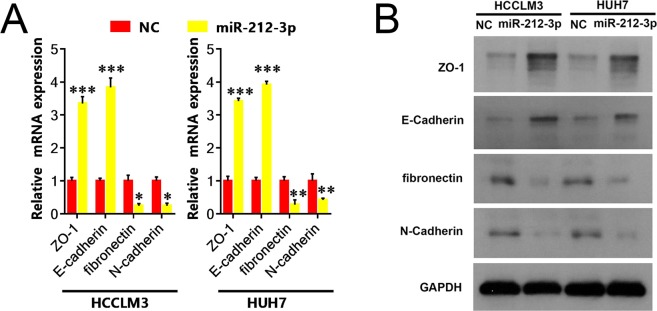
Upregulation of miR-212-3p alters the expression of EMT markers in hepatocellular carcinoma cells. qRT-PCR and western blot analysis were used to detect (**A**) mRNA and (**B**) protein expression of ZO-1, E-cadherin, and the mesenchymal markers fibronectin and N-cadherin in HCCLM3 or Huh7 cells. The results revealed that the mRNA and protein expression levels of ZO-1 and E-cadherin were upregulated in the miR-212-3p group compared to the control group, whilst the mRNA and protein expression of fibronectin and N-cadherin were downregulated in HCCLM3 or Huh7 cells treated with miR-212-3p compared to the control group. n = 3, *P < 0.05; **P < 0.01; ***P < 0.001.

We next assessed the effects of miR-212-3p on EMT. We measured the expression of epithelial ZO-1 and E-cadherin^15,16^, mesenchymal N-cadherin^17^ and fibronectin markers in Huh7 and HCCLM3 cells via qRT-PCR or western blotting. Figure 3A,B show that the upregulation of miR-212-3p led to substantially higher levels of E-cadherin and ZO-1 at the mRNA and protein level, whilst N-cadherin and fibronectin expression levels decreased in comparison to the control groups. We thus concluded that miR-212-3p inhibited cell invasion by modifying EMT markers in HCC cells.

### CTGF is directly targeted by miR-212-3p via the 3′-UTR

**Figure 4:**
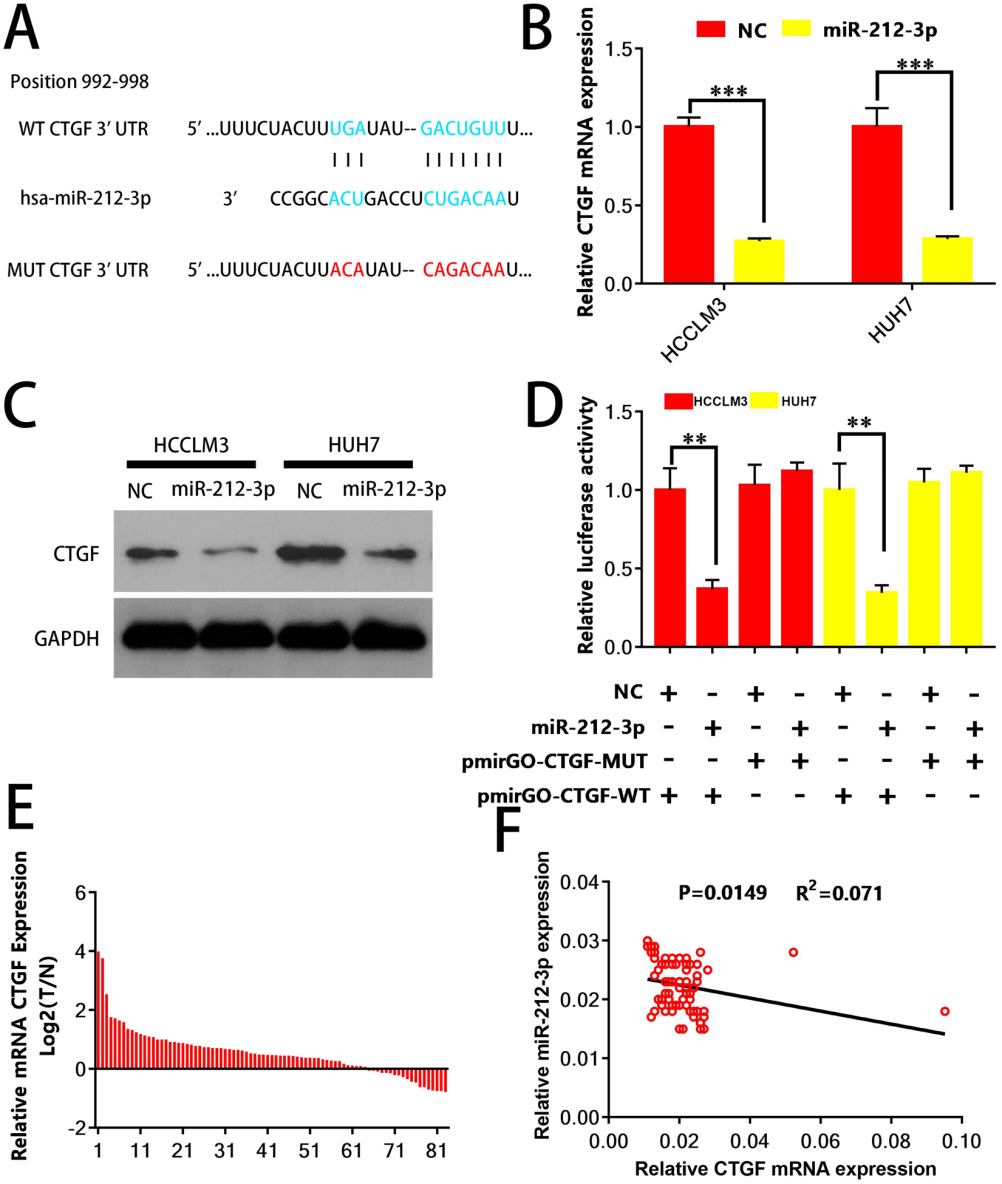
CTGF is directly targeted by miR-212-3p via the 3′-UTR. (**A**) Schematic of putative miR-212-3p binding sequences in the 3′-UTR of CTGF, and the generation of CTGF mutants. Relative (**B**) mRNA and (**C**) protein expression of CTGF in HCCLM3 or Huh7 cells with or without miR-212-3p were determined. (**D**) Luciferase assays were used to determine reporter activity in HCCLM3 or Huh7 cells co-transfected with either miR-212-3p mimics or NC, and either pmirGLO-CTGF-WT or pmirGLO-CTGF-MUT. (**E**) CTGF mRNA expression in the HCC samples was determined by qRT-PCR and (**F**) negatively correlated with miR-212-3p expression. *P < 0.05; **P < 0.01; ***P < 0.001.

An assumed miR-212-3p binding site in the 3′-UTR of connective tissue growth factor (CTGF) was revealed via TargetScan (http://www.targetscan.org/vert_72/). Luciferase reporter MUT 3′ UTR and CTGF WT 3′ UTR target sequences (Fig. 4A) were constructed to ascertain if miR-212-3p could directly bind to the CTGF 3′ UTR. CTGF expression was reduced in Huh7^miR-212-3p^ and HCCLM3^miR-212-3p^ cells (Fig. 4B,C) as confirmed via qRT-PCR and western blot analysis. As predicted, the overexpression of miR-212-3p inhibited CTGF 3′ UTR-wt luciferase reporter activity, compared to the CTGF 3′ UTR-mut in Huh7 and HCCLM3 cells (Fig. 4D). The expression of CTGF was upregulated in the majority of HCC samples (Fig. 4E), and an inverse-correlation with miR-212-3p expression was revealed by Spearman rank analysis (Fig. 4F, R^2^ = 0.071, P = 0.015). These results imply that miR-212-3p, through binding to the 3′-UTR of CTGF in Huh7 or HCCLM3 HCC cells, regulates CTGF expression.

### CTFG upregulation partially enhances the invasion ability of HCC cells

**Figure 5:**
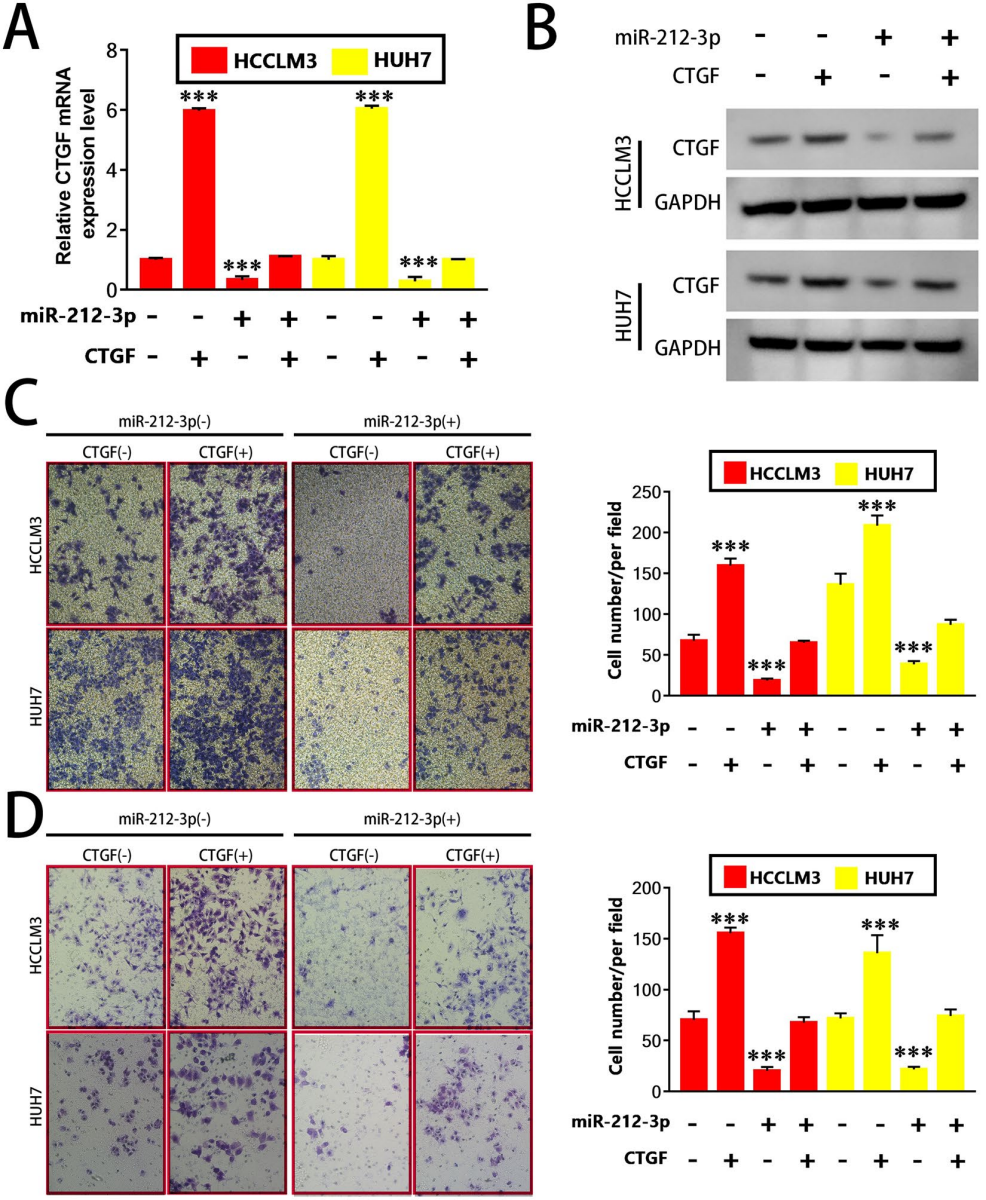
Up-regulation of CTGF partially enhances the invasion of HCC cells. CTGF overexpression can rescue the miR-212-3p-induced reduction in (**A**) mRNA and (**B**) protein expression in HCCLM3 and Huh7 cells. Migration (**C**) and invasion ability (**D**) of HCCLM3 or Huh7 cells was examined by Transwell assays. The over-expression of CTGF rescued the expression of miR-212-3p- and reduced migration and invasion. 200× magnification, n = 3, *P < 0.05; **P < 0.01; ***P < 0.001.

The functionality of miR-212-3p in HCC CTGF was validated through assaying Huh7^miR-212-3p^ and HCCLM3^miR-212-3p^ cells transfected with CTGF overexpression plasmids (Huh7^miR-212-3p+CTGF^ and HCCLM3^miR-212-3p+CTGF^), and the efficacy of the intervention was evaluated via western blot and qRT-PCR (Fig. 5A,B). CTGF overexpression led to a recovery of HCCLM3^miR-212-3p^ and Huh7^miR-212-3p^ invasion and migration ability (Fig. 5C,D) assessed via transwell assays. Thus, miR-212-3p suppresses CTGF expression, inhibiting HCC migration and invasion.

### Up-regulation of CTGF partially rescues the expression of EMT markers in HCC cells

**Figure 6:**
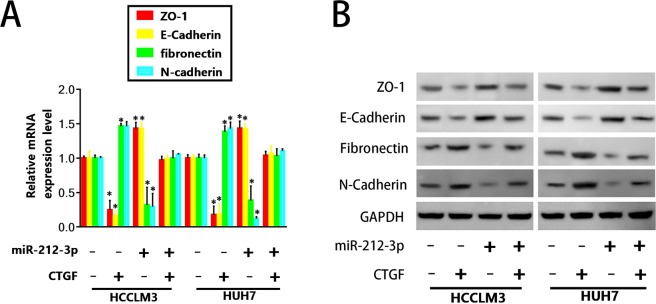
Up-regulation of CTGF partially rescues EMT marker expression in response to the upregulation of miR-212-3p. qRT-PCR and western blot analysis were used to determine the (**A**) mRNA and (**B**) protein levels of ZO-1, E-cadherin, fibronectin and N-cadherin in each group. (**C**) Schematic diagram of the possible mechanisms of miR-212-3p in HCC. n = 3, *P < 0.05; **P < 0.01; ***P < 0.001.

The upregulation of CTGF in Huh7 or HCCLM3 cells partially rescued EMT. We found that E-cadherin and ZO-1 were considerably down-regulated at both the mRNA and protein level in HCCLM3^miR-212-3p+CTGF^, compared to HCCLM3^CTGF^ cells, whilst N-cadherin and fibronectin levels appreciably recovered in HCCLM3^miR-212-3p+CTGF^, compared to HCCLM3^CTGF^ (Fig. 6A). These results were comparable in Huh7 cells (Fig. 6B). These results indicate that miR-212-3p influences the expression of EMT markers through its ability to downregulate CTGF.

## Discussion

Recurrence and metastasis contribute to the mortality of HCC patients following post-surgical resection. Understanding the metastatic mechanisms of HCC are required for effective HCC therapeutics. Numerous analyses have validated the role of miRNAs in HCC progression and metastasis^17–20^ yet their primary molecular mechanisms remain poorly understood. Further studies on the regulatory characteristics of miRNAs during HCC progression and metastasis are required to assess their potential as prospective clinical diagnostic and prognostic biomarkers.

We found that miR-212-3p is downregulated in HCC cells and tissues, and correlates with vascular invasion (p = 0.001) and the absence of capsule formation (p = 0.009). Low miR-212-3p expression was related to lower survival rates and miR-212-3p was found to target CTGF. We found that miR-212-3p could repress HCC cell proliferation, migration, and invasion, by inhibiting CTGF and reducing EMT. We therefore established miR-212-3p as a HCC biomarker and potential therapeutic target.

A series of complex events occur during tumor metastasis^21^. The invasive-metastatic cascade involves EMT endowing non-invasive tumor cells with metastatic ability, enabling their migration into the extracellular matrix, the ability to cross the basal membranes of vessels, to persist in the bloodstream, and initiate clonal metastatic growth on other organs via angiogenesis^22^. EMT promotes hepatic fibrosis in a pre-metastatic microenvironment^23^. Several analyses have confirmed the influence of miRNAs in the EMT process^24,25^. In this study, miR-212-3p positively correlated with E-cadherin and ZO-1, and negatively correlated with N-cadherin and fibronectin. *In vitro* trials also established that miR-212-3p can reverse the metastatic capabilities of HCC cells and the EMT phenotype.

CTGF is vital to both angiogenesis and EMT due to its role as a transcriptional TGF-β target and ability to interact with extracellular matrix and cell surface proteins, in addition to other growth factors^26^. TGF-β is pro-metastatic in colorectal cancers (CRC) and is related to a poor outcome^27^. Similarly, CTGF correlates with poor prognosis^28^. TGF-β triggers CTGF expression via the canonical pSMAD2/3 pathway^29^. CTGF also regulates KLF4 to trigger EMT in Kawasaki disease^30^. CTGF enhances ANGPT2 expression, a key element in tumor angiogenesis^31^. The suppression of CTGF expression appreciably decreases angiogenesis^31^ and tumor growth^32^ *in vivo*. We found that CTGF is a direct miR-212-3p target via dual luciferase assays. The HCC cell lines overexpressing miR-212-3p displayed considerably lower CTGF expression at the mRNA and protein level. CTGF overexpression rescued the migration and invasion of Huh7 and HCCLM3 cells overexpressing miR-212-3p. Additionally, CTGF up-regulation partly rescued the miR-212-3p-induced variations in EMT markers. These results confirm that CTGF-mediated EMT marker alterations are induced by miR-212-3p inhibition. This highlights the regulatory miR-212-3p/CTGF axis as a novel therapeutic target during HCC progression.

In summary, miR-212-3p was down-regulated in HCC cells and tissue and correlated to tumor encapsulation and vascular invasion. The effects of miR-212-3p on HCC cells were dependent on its ability to downregulate CTGF-mediated EMT markers. The miR-212-3p/CTGF axis thus represents a promising molecular target during HCC treatment.
